# Identification and Characterization of Genetic Determinants of Isoniazid and Rifampicin Resistance in *Mycobacterium tuberculosis* in Southern India

**DOI:** 10.1038/s41598-019-46756-x

**Published:** 2019-07-16

**Authors:** Asma Munir, Narender Kumar, Suresh Babu Ramalingam, Sembulingam Tamilzhalagan, Siva Kumar Shanmugam, Alangudi Natarajan Palaniappan, Dina Nair, Padma Priyadarshini, Mohan Natarajan, Srikanth Tripathy, Uma Devi Ranganathan, Sharon J. Peacock, Julian Parkhill, Tom L. Blundell, Sony Malhotra

**Affiliations:** 1 Department of Biochemistry, University of Cambridge, Tennis Court. Rd., Cambridge, CB2 1GA UK; 2 Department of Medicine, University of Cambridge, Hills Rd., Cambridge, CB2 0QQ UK; 3 ICMR-National Institute for Research in Tuberculosis, Chennai, 600031 India; 4 London School of Hygiene & Tropical Medicine, Keppel Street, London, WC1E 7HT UK; 5 Wellcome Trust Sanger Institute, Hinxton, Cambridgeshire CB10 1SA UK; 6 Present Address: Birkbeck College, University of London, Malet Street, WC1E7HX London, UK

**Keywords:** Mutation, Molecular modelling, Protein function predictions

## Abstract

Drug-resistant tuberculosis (TB), one of the leading causes of death worldwide, arises mainly from spontaneous mutations in the genome of *Mycobacterium tuberculosis*. There is an urgent need to understand the mechanisms by which the mutations confer resistance in order to identify new drug targets and to design new drugs. Previous studies have reported numerous mutations that confer resistance to anti-TB drugs, but there has been little systematic analysis to understand their genetic background and the potential impacts on the drug target stability and/or interactions. Here, we report the analysis of whole-genome sequence data for 98 clinical *M. tuberculosis* isolates from a city in southern India. The collection was screened for phenotypic resistance and sequenced to mine the genetic mutations conferring resistance to isoniazid and rifampicin. The most frequent mutation among isoniazid and rifampicin isolates was S315T in *katG* and S450L in *rpoB* respectively. The impacts of mutations on protein stability, protein-protein interactions and protein-ligand interactions were analysed using both statistical and machine-learning approaches. Drug-resistant mutations were predicted not only to target active sites in an orthosteric manner, but also to act through allosteric mechanisms arising from distant sites, sometimes at the protein-protein interface.

## Introduction

Tuberculosis (TB) caused an estimated 1.3 million deaths worldwide in 2016 (WHO Global Tuberculosis Report, 2017). The major challenge in the treatment of tuberculosis is the emergence of drug-resistant *Mycobacterium tuberculosis*^1^. The drugs available for tuberculosis treatment are categorised into first-line (isoniazid (INH), rifampicin (RIF), pyrazinamide (PZA), ethambutol (EMB) and streptomycin (STR)) and second line (including fluoroquinolones, thioamides, cycloserine and the injectable aminoglycosides). Rising rates of multi-drug-resistant tuberculosis (MDR-TB, defined as resistance to INH and RIF), are of immense concern for TB control worldwide. Extended treatment is required with multiple drugs that have a higher rate of side effects but limited rate of treatment success (Gygli *et al*.^2^). India accounts for the highest burden of tuberculosis globally and also ranks top among the countries for MDR-TB cases (WHO Global Tuberculosis Report, 2017).

Drug resistance arises mainly from spontaneous mutations in the bacterial genome. Resistance to first-line anti-TB drugs has been linked to mutations in *katG*^3^ and *inhA*^4^ for INH resistance; *rpoB* for RIF resistance^5^; *embB* for EMB resistance^6^; *pncA* for PZA resistance^7,8^ and *rpsL* and *rrs* for STR resistance^9,10^. Several large scale whole genomic studies have identified a catalogue of resistance-associated mutations^11–16^. Molecular methods such as Xpert MTB/RIF^17^ and Line Probe Assay (Cepheid, Sunnyvale, CA, USA) rely on these genetic determinants to identify drug-resistance. The predictive accuracies of known mutations for INH and RIF are comparable to the current gold standard of phenotypic drug susceptibility tests(DSTs), but vary for other drugs^18^.

Understanding and tackling drug resistance may be informed by an understanding of bacterial population structure. Fenner *et al*. (2012) showed that lineage was associated with particular mutations and variable levels of drug resistance, and suggested a role for epistatic interactions between mutations and genetic background in resistant strains^19^. In addition, some lineages such as L2 (Beijing strains) have an increased frequency of drug resistance^20^. India is notable for having a predominance of *M. tuberculosis* lineage 1 in the South and lineage 3 in the North^21^. The contributions of these lineages in worldwide studies have been limited compared to globally-dominant lineages 2 and 4. Recently, Manson *et al*. (2017) reported that known mutations for INH and RIF accounted for only 72% of resistance among strains from Chennai, India, necessitating more studies to catalogue resistance-associated mutations^22^. Therefore, studies are needed to understand the epidemiology and generate a comprehensive list of resistance-conferring mutations. Since then the effects of mutations have been reported for isolates representing the global diversity arising from four lineages of *M. tuberculosis*^23^.

The gene mutations in *M. tuberculosis* that are associated with drug resistance result in the alteration of the phenotype due to changes in drug-bacterial interactions, including protein stability and/or structural changes that interfere with the mechanism of drug action. Detailed insights into mechanisms of drug-resistance mutations can help in the design of new and improved existing drugs, the selection of improved drug targets and even identification of new drug targets. Because elucidating the effects of mutations experimentally is expensive and time consuming, many efforts have been made to develop computational methods that can predict the effects of mutations. These can be trained either using protein sequences alone or by taking structural features of the proteins into account. The most commonly used sequence-based methods such as SIFT^24^ and PolyPhen^25^ make use of features such as sequence conservation, estimated from multiple sequence alignments derived from closely related sequences, to predict the effects of mutations on protein function, while the structure-based methods benefit from the use of extensive structural parameters from the available 3D structures of proteins. Some of the well-established structure-based prediction programs include PoP-MuSiC^26^, BeAtMuSiC^27^, Site-Directed Mutator (SDM)^28–30^ and mutation Cut-off Scanning Matrix (mCSM)^31–33^.

SDM uses a statistical approach to predict the effects of mutations on protein stability and is based on the analysis of naturally occurring amino-acid substitutions expressed in the form of environment-specific substitution tables (ESSTs). SDM calculates a stability score, which is analogous to the free-energy difference between the wildtype and the mutant protein. mCSM is based on a machine-learning approach and uses graph-based signatures to predict the effects of mutations on protein stability. mCSM has been trained to predict the effects of mutations on protein stability (mCSM-stability), protein-protein interactions (mCSM-PPI), protein-ligand affinity (mCSM-lig) and protein-nucleic acid affinity (mCSM-NA). Our group has also developed tools, Intermezzo (Ochoa B. and Blundell T.L., unpublished) and Arpeggio^34^, to visualise the interactions of small-molecule drug binding; these go beyond hydrogen bonds and lipophilic interactions to include directional pi–pi and other interactions of aromatic groups, as well as interactions mediated by drugs containing halogens. These programs have already been used by our group and others to generate insights into mutations in many genetic diseases, including cancer^35,36^.

In this study, we discuss the genome sequences of 98 *M. tuberculosis* isolates from Chennai in India. We define the population structure and map mutations onto the structures of their drug targets (*katG, inhA* and *rpoB*). The impacts of these mutations on protein structure stability, protein-protein and protein-ligand interactions are predicted using SDM and mCSM.

## Results

### Lineage diversity and phenotypic resistance

**Figure 1:**
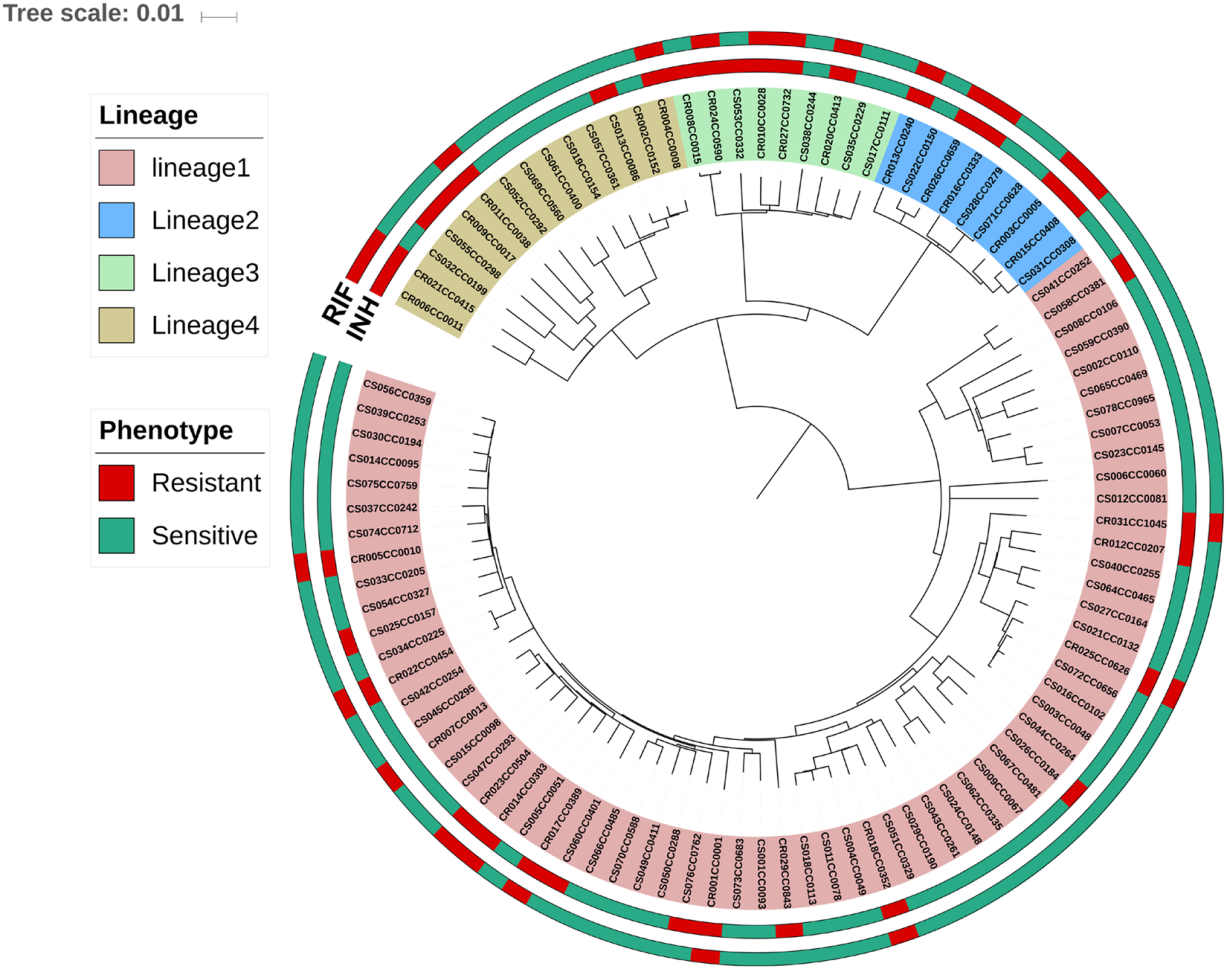
Whole genome SNP based phylogenetic of 98 isolates. The circles 1 and 2 starting from inside out represent resistance and susceptibility to INH and RIF, respectively.

An SNP-based phylogenetic tree was constructed based on the sequence data of the 98 study isolates. This demonstrated that lineage 1 (Indo-Oceanic lineage) predominated (66, 67%), with the remainder falling into lineages 2 (9, 9%), 3 (9, 9%) and 4 (14, 15%) (Fig. 1). Phenotypic drug resistance to INH and RIF was mapped across these 98 isolates. Thirty-four and 24 isolates were resistant to INH and RIF, respectively. Resistant isolates were present across all four lineages (Fig. 1). All INH-resistant isolates in lineage 2 were also resistant to RIF, while isolates in other lineages (seven in lineage 1, two in lineage 3 and four in case of lineage 4) were INH resistant but RIF susceptible.

### Genetic determinants of resistance

**Figure 2:**
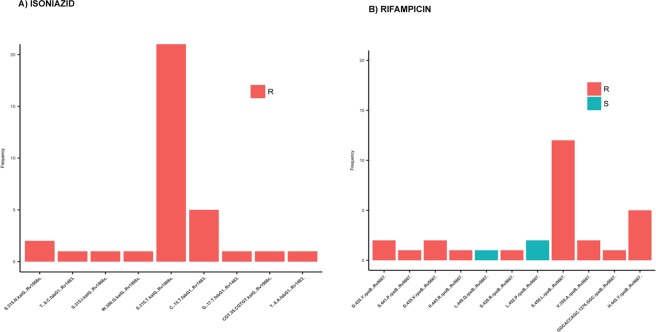
Frequency of resistance mutations identified in 98 isolates. The colours show phenotypic results for (**A**) isoniazid and (**B**) rifampicin.

The genetic basis for INH resistance was determined through an analysis of resistance-conferring mutations in *katG*, *fabG*1 promoter region and *inhA*. Thirty-three of the 34 resistant isolates had known resistance mutations either in *katG* and/or *fabG*1 promoter or both. The remaining isolate had no known resistance-conferring mutation in *katG* or *fabG*1 promoter, but a stop-codon mutation was noted at the 575^th^ codon in *katG* that resulted in premature termination and hence protein truncation, which might lead to the resistant phenotype. The S315 T mutation in *katG* and C-15T mutation in *fabG1* promoter region were the most common mutations in resistant isolates, with frequencies of (23/34) 67.6% and (5/34) 14.7%, respectively (Fig. 2). Together, these two mutations accounted for 82% of INH resistance, which is concordant with previous reports^37,38^. We noted several other known mutations in *katG* and *inhA* in a minority of INH-resistant isolates (Table 1). One INH-resistant isolate possessed both S315T *katG* and C-15T *fabG1* mutations. Resistant isolates have been reported previously to acquire multiple mutations in the same resistance-causing gene^39^. Five of the INH-resistant isolates had two mutations while one isolate had three mutations in *katG*.

**Table 1:** Mutations identified in phenotypically isoniazid resistant strains.

Drug	Gene	Mutation	Frequency
ISONIAZID	*katG*	S/315/T	23
		S/315/N	2
		S/315/I	1
		W/300/G	1
		S/575/Stop	1
		In-del	1
	*fabG1*	C/-15/T	5
		T/-8/A	1
		T/-8/C	1
		G/-17/T	1
	*inhA*	I/194/T	3
		I/21/T	1
		S/94/A	1

The genetic basis for RIF resistance was determined through an analysis of resistance-conferring mutations in *rpoB*^40^. Twenty-two isolates (92%) contained known resistance-conferring mutations in *rpoB* (Table 2). S450L and H445Y were the most frequent of these, with a frequency of 52% and 26%, respectively (Fig. 2). For the remaining two isolates where no resistance conferring mutations were identified, one isolate had a deletion in the RRDR (Rifampicin-resistance determining region), which is known to confer resistance^41^, but the other isolate had wildtype *rpoB*. Several other known *rpoB* mutations for RIF resistance were present at low frequency among the RIF resistant isolates (Table 2). Compensatory mutations have been reported to occur in *rpoC* (23208709), which were also identified in a fraction of RIF-resistant isolates (Table 2).

**Table 2:** Mutations identified in phenotypically rifampicin resistant strains.

Drug	Gene	Mutation (codon change)	Frequency
RIFAMPICIN	*rpoB*	S/450/L (TCG/TTG)	12
		D/435/Y (GAC/TAC)	1
		D/435/V (GAC/GTC)	2
		H/445/R (CAC/CGC)	1
		H/445/Y (CAC/TAC)	5
		S/428/R (AGC/AGG)	1
		V/359/A (GTG/GCG)	2
		S/441/P (TCG/CCG)	1
		L/452/P (CTG/CCG)	2
		L/449/Q (CTG/CAG)	1
		In-del	1
	*rpoC*	L/516/P (CTG/CCG)	1
		V/483/G (GTG/GGG)	2
		I/491/T (ATC/ACC)	1
		N/416/T (AAC/ACC)	1

### Modelling mutations in katG

*katG*, a heme-dependent enzyme with dual catalase and peroxidase activity, is involved in the activation of the pro-drug, isoniazid^42^. The mutations listed in the Table 1 were mapped on the crystal structure of *katG* (PDB ID: 1SJ2) to visualize their locations on the protein (Supplementary Fig. S1). This showed that mutations were located in the N-terminal domain of the protein near the heme group. The effects of mutations on protein stability were predicted by SDM and mCSM (Table 3).

**Table 3:** Predicted effects of mutations in katG, inhA, rpoC and rpoB on protein stability (using SDM2, mCSM), protein-protein interactions (using mCSM-PPI) and protein-ligand interactions (using mCSM-lig). All ΔΔG values are in kcal/mol.

Target	Mutation	Predicted ΔΔG SDM2	Predicted ΔΔG mCSM	Predicted Affinity Change mCSM-lig	Predicted ΔΔG mCSM-PPI
*katG*	S315T	0.34	−0.33		−0.065
	S315I	0.25	−0.424		0.06
	S315N	−0.02	−0.184		−0.203
	A106V	−1.14	−0.234		−1.625
	W300G	0.42	−3.804		−0.521
*inhA*	S94A	0.36	−0.89	−0.485	−0.14
	I194T	−2.42	−2.347	−1.603	−0.423
	I21T	−1.06	−1.84	−1.679	−0.107
*rpoB*	S450L	0.14	−0.152	−0.234	−0.215
	H445R	−1.18	−1.583	−0.633	−1.362
	H445Y	−0.43	−0.075	−0.173	−0.157
	D435Y	−0.71	0.042	−0.138	−0.294
	D435V	0.33	1.513	−0.193	0.155
	L452P	0.58	−0.982	−1.007	−0.018
	V359A	−2.36	−1.849	−0.256	−0.426
	S428R	0.26	−0.356	−0.736	−0.203
*rpoC*	L516P	−3.918	−1.48		−0.401
	N416T	−0.06	−0.194		−1.086
	V483G	−2.72	−3.134		−0.594
	I491T	−1.51	−2.803		−0.464

#### S315T, S315I, and S315N

**Figure 3:**
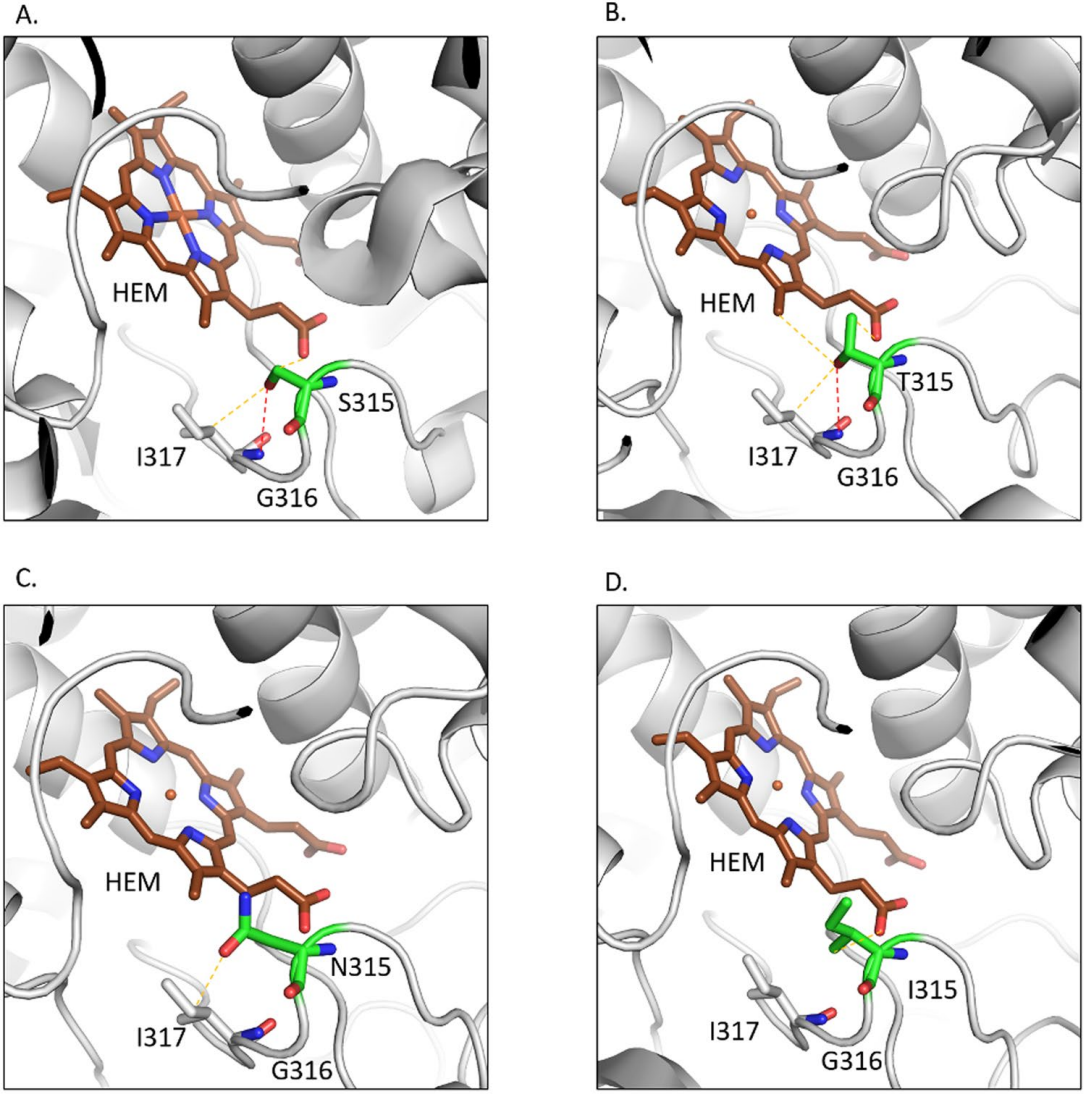
(**A**) The wildtype residue S315 of katG forms a hydrogen bond (shown as red dotted lines) with the main chain of I317 and a weak hydrogen bond (orange dotted lines) with heme and sidechain of I317. (**B**) The mutant residue T315 gains an additional weak hydrogen bond and hydrophobic interactions with heme. (**C**) The mutant residue loses the hydrogen bond with I317 and a weak hydrogen bond with heme. (**D**) The hydrophobic sidechain of isoleucine retains the weak hydrogen bond with RFP and gains hydrophobic interaction with heme.

The residue S315 is located at the narrowest edge of a substrate access channel that connects heme to the molecular surface (Fig. 3A). The substitution of serine to a bulkier sidechain threonine (Fig. 3B) has been described previously to constrict the channel and limit accessibility to heme^23,43^. Other substitutions of serine to isoleucine and asparagine, reported previously, were present in our clinical isolates. We predicted the effects of these mutations using SDM and mCSM and analysed the interatomic interactions of the wildtype and the mutant residues using intermezzo. The sidechain of the wildtype residue S315 was observed to form a hydrogen bond with the mainchain of I317 and a weak hydrogen bond with the heme and the sidechain of I317 (Fig. 3A). When mutated to threonine, the sidechain gains a weak hydrogen bond and hydrophobic interactions with heme (Fig. 3B). The substitution of S315 to asparagine results in the loss of a hydrogen bond with I317 and a weak hydrogen bond with the heme (Fig. 3C). When mutated to isoleucine, the mutated residue loses both the hydrogen bond and the weak hydrogen bond with I317 and gains hydrophobic interactions with heme (Fig. 3D). Hence, substitution of S315 by other residues results in weaker affinity of *katG* towards heme.

#### W300G

**Figure 4:**
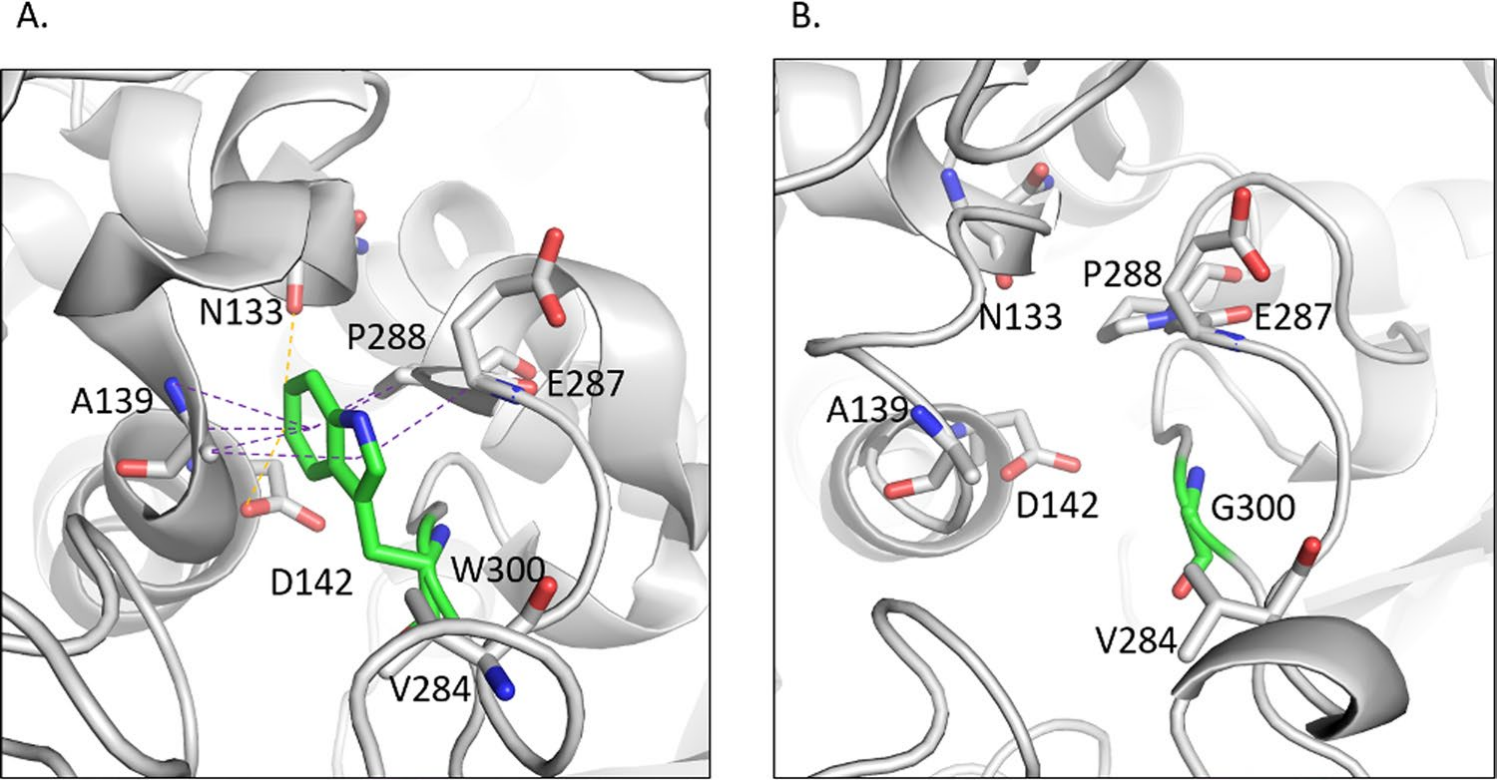
(**A**) The katG wildtype residue W300 is involved in the formation of carbon-pi interactions with A139, E287 and P288 and donor-pi interactions with A139. All the atom-pi interactions are shown as purple dotted lines. It also forms weak hydrogen bonds with N133 and D142 indicated as orange dotted lines. (**B**) The mutant residue G300 loses all the interactions with the surrounding residues.

The wildtype residue W300 makes carbon-pi interactions with A139, E287 and P288 and donor-pi interactions with A139 (Fig. 4A). It makes hydrophobic contacts with A139, E287, P288 and V284 and is involved in forming weak hydrogen bonds with D142 and N133. The substitution of tryptophan by glycine causes the loss of all the interactions made by the wildtype residue (Fig. 4B).

Overall, the mutations in katG are predicted to be slightly destabilising or near neutral which implies that they do not come with a greater fitness cost for the protein. It is very likely the mutations in katG interfere with the drug binding of the protein.

### Modelling mutations in *inhA*

We mapped the INH-resistance mutations identified onto the homo tetramer structure of *inhA* (PDB ID: 1ZID) (Supplementary Fig. S2). The mutations were found to be located around the drug-binding pocket (the drug INH is shown in yellow, Supplementary Fig. S2). The effects of mutations on protein stability and ligand affinity were predicted using SDM, mCSM and mCSM-lig, respectively. The predictions made by the two programs are given in Table 3. These mutations are predicted to decrease the affinity of the drug towards the protein, hence resulting in a drug resistance phenotype.

#### I194T and 121T

The wildtype residue I194T is also present close to the INH-NAD-adduct binding site and is observed to form hydrophobic contacts with T196 and W230. The substitution of isoleucine by residues with shorter and polar sidechains results in the loss of hydrophobic interactions with W230 and hence decrease of the affinity of INH binding to *inhA* (Supplementary Fig. S3A,B). I194T is also reported to be present in ethionamide-resistant strains^44^. The wildtype residue I21 is present close to the INH-NAD adduct binding pocket and is involved in making hydrophobic contacts with M147 and V238. The substitution of isoleucine to a polar residue threonine results in the loss of these hydrophobic interactions and hence affects the binding of INH-NAD adduct to *inhA* (Supplementary Fig. S3C,D).

### *rpoB* subunit of RNA polymerase

**Figure 5:**
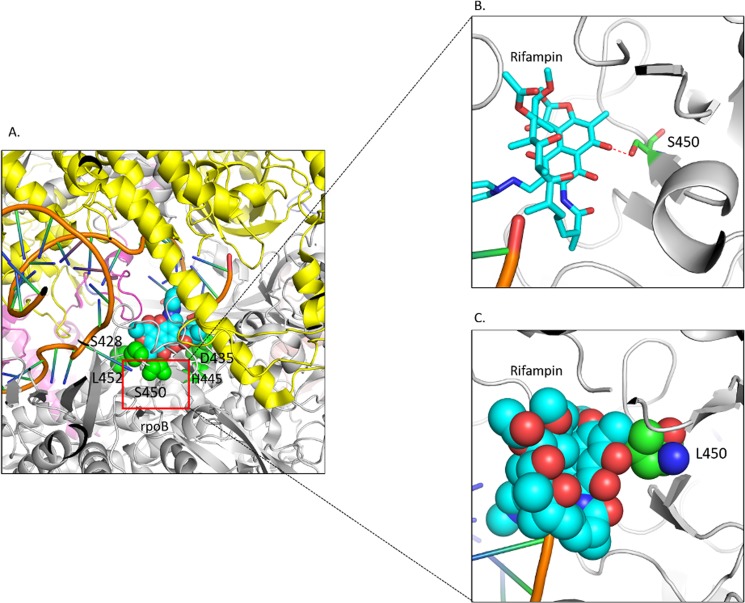
(**A**) Mutations mapped on the crystal structure of rpoB. Mutations mapped in green spheres are located in the rifampicin (cyan spheres) binding pocket. (**B**) The wildtype residue S450 (green) forms hydrogen bond (shown in red dotted lines) with RFP. (**C**) The bulkier sidechain of mutant L450 causes steric clashes with RFP.

*rpoB* is the β subunit of the bacterial RNA polymerase, which is targeted by the first-line anti-tuberculosis drug, rifampicin. Resistance to rifampicin is caused by the mutations in the binding pocket of the drug. The effects of mutations were predicted using SDM2 and mCSM and the interatomic interactions analysed by Intermezzo. Whereas a previously reported analysis^23^ was based on a homology model, we were able to analyse the impacts of the mutations in the *rpoB* and *rpoC* subunits of the RNA polymerase using a recently defined crystal structure (PDB ID: 5UHC)^45^ mapped onto the highly conserved drug binding pocket (Supplementary Fig. S4 and Fig. 5A).

#### S450L

S450L is one of the most frequently occurring RFP-resistant mutations^46,47^. It is predicted to decrease the affinity of the drug towards the protein by mCSM-lig. Evaluation of interatomic interactions of the wildtype and the mutant residue using Intermezzo showed that the wildtype residue S450 forms a hydrogen bond with rifampicin (Fig. 5B) but when it is mutated to leucine, the longer sidechain causes steric clashes with the drug (Fig. 5C), reducing its affinity towards the drug as indicated by mCSM-lig value (Table 3).

#### H445R and H445Y

H445R was observed to be present in the RFP-binding pocket and the interatomic analysis using Intermezzo revealed that this mutation affects the interactions with the surrounding residues (Supplementary Fig. S5A). The wildtype residue forms hydrophobic interactions with D435, which are involved in forming hydrophobic interactions with RFP, while the mutant residue R445 causes steric clashes with D435 (Supplementary Fig. S5B). The wildtype histidine also forms carbon-pi contacts with V168 and R448 and cation-pi contacts with R448, which are lost for the mutant R445 and Y445. The mutant residue Y445 gains the carbon-pi and cation-pi contacts with D435 (Supplementary Fig. S5C).

#### D435Y and D435V

**Figure 6:**
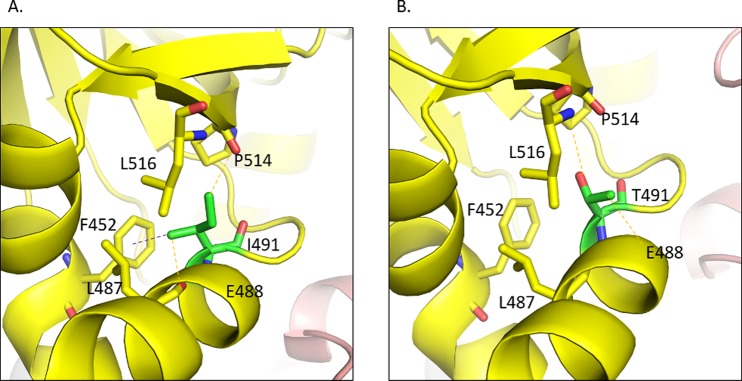
(**A**) The wildtype residue D435 in rpoB forms salt bridges with R448 and R607 (blue dotted lines), weak hydrogen bonds (orange dotted lines) with R607 and RFP and hydrophobic interactions with RFP and H445. (**B**) The mutant V435 loses the ionic interactions and the weak hydrogen bonds. (**C**) The mutated residue Y435 causes steric clashes with RFP. (**D**) Spheres representation of the steric clash between Y435 and RFP.

The wildtype residue D435 is located within 3.1 Å and forms a salt bridge with R448 and R607 (Fig. 6A). It also forms a weak hydrogen bond with R607 and rifampicin, and hydrophobic interactions with H445 and rifampicin. The substitution of aspartic acid to valine causes the loss of ionic interactions with R448 and R607 (Fig. 6B). The mutated residue also loses its weak hydrogen bond with rifampicin. The mutation of aspartic acid to tyrosine introduces a steric clash between the tyrosine sidechain and the drug (Fig. 6C,D), leading to the decreased affinity of the drug towards the protein as indicated by mCSM-lig values (Table 3). Other mutations were also observed to alter the interactions with the drug as in L452P (Supplementary Fig. S6) and with the surrounding residues in the case, V359A (Supplementary Fig. S7), and S428R (Supplementary Fig. S8).

### *rpoC* subunit of RNA polymerase

We observed four mutations in the *rpoC* subunit in response to rifampin (I491T, L516P, N416T, V483G), which are present at the interfaces with other subunits in the RNA polymerase assembly (Supplementary Fig. S9). The mutations L516P, I491T and V483G are in close proximity with the subunits *rpoA* and *rpoZ*, while the mutation N416T is located at the interface with the subunit *rpoB*. The effects of these mutations on protein-protein interactions were predicted using mCSM-PPI (Table 3).

**Figure 7:**
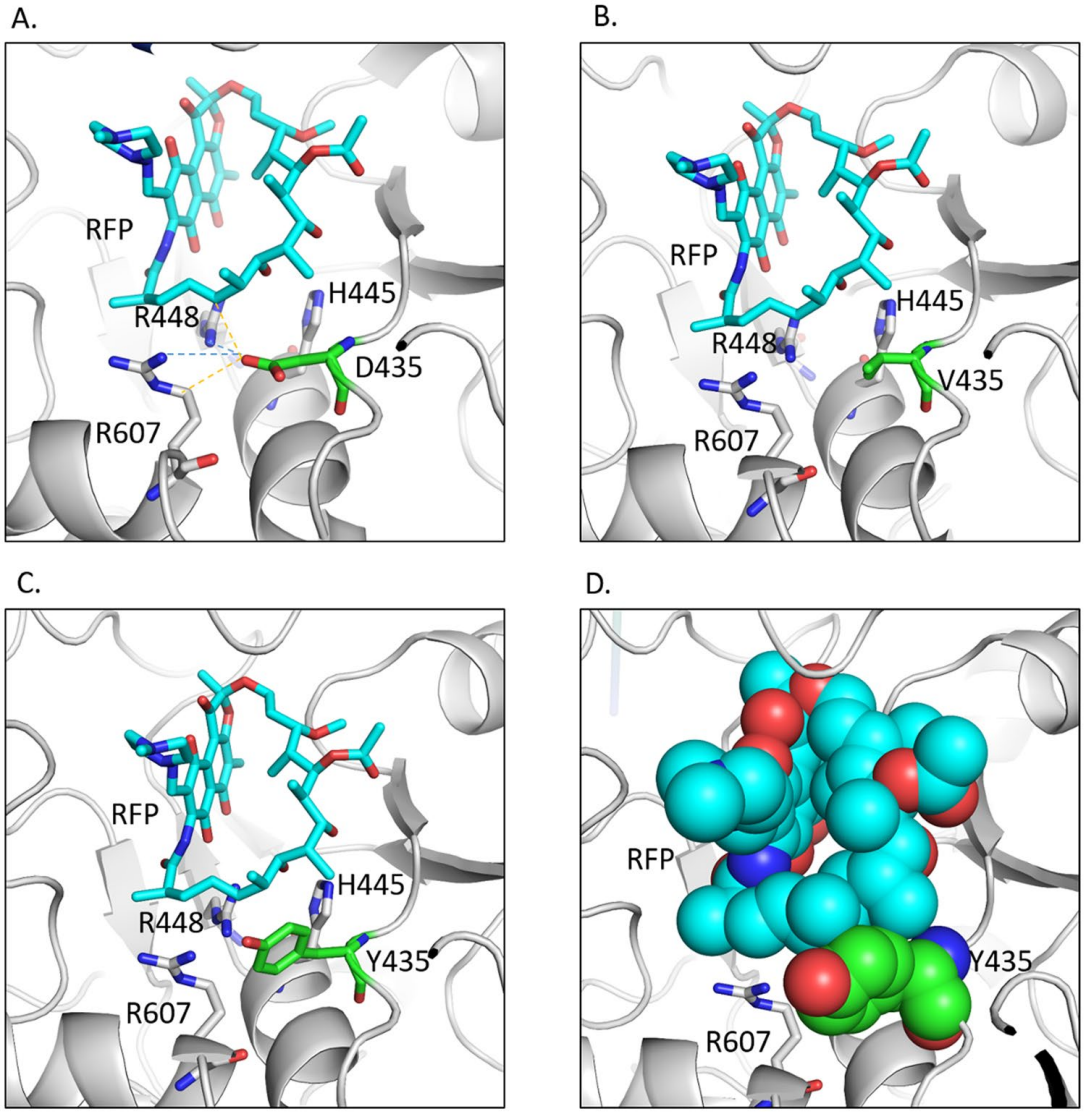
(**A**) The *rpoC* wildtype residue I491 makes a carbon-pi interaction with F452. It also forms a weak hydrogen bond with the backbone of L487 and another weak hydrogen bond with the carbonyl oxygen of P514. (**B**) The mutated residue loses the carbon-pi interaction with F452 and makes a weak hydrogen bond with the sidechain CG of the residue P514 and another weak hydrogen bond with the backbone of E488.

The wildtype residue I491 is involved in making hydrophobic contacts with L487, L516, P514 and F452 (Fig. 7A). It also forms a carbon-pi contact with F452 and weak hydrogen bonds with the carbonyl oxygen of the residues P514 and L487. When mutated to threonine, the carbon-pi and hydrophobic contacts are lost. Moreover, the mutated residue loses the weak hydrogen bond with L487 and gains a weak hydrogen bond with the mainchain carbonyl oxygen of E488 (Fig. 7B). It also loses the weak hydrogen bond with the carbonyl oxygen of P514 and instead gains a weak hydrogen bond with the sidechain carbon CG of the P514. The wildtype residue L516 is involved in making hydrophobic contacts with I491, L487 and E488. When mutated to proline, the bulkier sidechain of proline causes a steric clash with I491 (Supplementary Fig. S10). For N416T, the wildtype asparagine forms a hydrogen bond and a weak hydrogen bond with R412 but when mutated to threonine it gains an additional hydrogen bond with R412. Threonine also gains a weak hydrogen bond with S1120 and hydrophobic interactions with T1053 from chain C (Supplementary Fig. S11). The wildtype residue V483 is involved in making hydrophobic contacts with W484, L449, V476 and L460, and makes a carbon-pi contact with W484. When mutated to glycine, it loses all the interactions with the surrounding residues (Supplementary Fig. S12).

Most of the mutations in *inhA* and *rpoB* affect both the stability and the drug-binding affinity of the protein. The mutations in *rpoC* are predicted to have an impact on both protein stability and protein-protein interactions.

## Discussion

Early detection of drug-resistant *M. tuberculosis* is key to rapid, effective patient treatment and reduction in ongoing transmission. Whole genome sequencing offers the potential to detect genetic mutations associated with phenotypic resistance at an earlier timepoint than culture-based methods, but the majority of *M. tuberculosis* genomes on which predictions are based belong to the globally dominant lineages 2 and 4. More studies are needed from regions where other lineages predominate, including in India where lineage 1 and 3 prevail. Here, we report the results of a study of whole genome sequencing of *M. tuberculosis* from Southern India, in which phenotypic DST was compared with sequence-based predictions to understand the frequency of already known and putative resistance-conferring mutations in this population. Our genome analysis and identification of previously known mutations conferring resistance to INH and RIF were extended by *in silico* analyses to gain insights into drug resistance mechanisms by mapping mutations onto the protein structure.

Lineage 1 was dominant in our collection, which is consistent with previous reports from the region (Singh *et al*. 2015). As *katG* is required for the activation of INH, mutations in *katG* are likely to be a first step in acquiring drug resistance and most mutations leading to resistance to INH are reported to be present in *katG*. The substitution S315T found here is the most frequent isoniazid-resistant mutation in several studies^48–50^. This has also been reported experimentally to be sufficient alone to confer resistance to INH^51^ and decrease the affinity of INH towards the protein^52^.

S94A substitution in *inhA* is a frequently reported mutation and has been well characterised in the literature. The analysis of the crystal structure of the mutant S94A by various groups^53–55^ indicates that the resistance mutation causes a reduced affinity of NADH binding to *inhA*, by disrupting the hydrogen-bonding network of a conserved water molecule in the active site by mutating the polar residue serine to the non-polar residue, alanine. These studies suggest that NADH binding is a pre-requisite for NAD-INH adduct formation and a decrease in binding affinity of NADH in mutants leads to drug resistance^53^. Furthermore, thermodynamic studies^56^ suggested no significant change in the adduct binding affinity to wildtype and mutant enzymes and they proposed that the resistance might be due to allosteric effects caused by the interaction of other proteins with *inhA* within the FAS II pathway. They also presented evidence to suggest that NAD-INH adduct forms in solution and the decrease in NADH affinity does not significantly alter the NAD-INH affinity for the enzyme^56^.

Drug-resistant mutations in *rpoB* have mostly been found in the 81-bp region of *rpoB*, sometimes called the Rifampicin Resistance Determining region (RRDR)^57^. Many mutations in *rpoB* occur in the highly conserved residues in the drug-binding pocket. These mutations in *rpoB* come with a fitness cost, while mutations in *rpoC* are proposed to compensate for this fitness cost^58^. de Vos *et al*. reported the presence of putative compensatory *rpoC* mutations in 23.5% of all rifampicin resistant isolates and these were associated with specific strain genotypes and specific *rpoB* mutation (S531L) as 44.1% of isolates with this mutation also harboured *rpoC* mutations^59^. A more recent study by Yun *et al*. (2018) reported the presence of *rpoC* mutations in 25.8% of the total isolates^60^. In our study, we found that 8/24 resistant isolates had mutations in *rpoC*, of which 7 had the S450L dominant mutation. This concurs with a previous study linking the dominant mutation with the acquisition of potential compensatory mutations. Moreover, the mutations in *rpoC* were found to be located at the interfaces with other subunits, as reported in earlier studies^58,59^.

An interesting observation from our study is that both the mutations S450L in *rpoB* and S315T in *katG* that dominated resistant isolates at no/low fitness cost^61–63^ are also predicted to have a near neutral effect on protein stability and/or ligand binding affinity (Table 3). This also supports the observation that frequency of occurrence of a drug-resistant mutation inversely correlates with fitness cost^64^.

Computational approaches such as ours for elucidating the effects of mutations are useful for the rapid analysis of a large dataset of mutations as experimental approaches may be time consuming and expensive. This analysis should be useful in understanding the mechanism of drug resistance in tuberculosis.

## Methods

### Bacterial isolates

The 98 *M. tuberculosis* isolates included here were from patients recruited at the Government Hospital for Thoracic Medicine, Chennai, India. Patients were diagnosed and treated under India’s RNTCP (Revised National Tuberculosis Control Program). Two cohorts were recruited, as follows:

(i) Cohort 1 (drug resistant tuberculosis) comprised adults with newly diagnosed drug resistant pulmonary TB (PTB), who were given RNTCP Category IV treatment (either LPA positive for RIF resistant *M. tuberculosis* or GeneXpert positive with Rifampicin resistance at entry)
(ii) Cohort 2 (drug sensitive tuberculosis) comprised adults with newly diagnosed active PTB, who were given RNTCP Category I treatment (sputum smear positive for acid fast bacilli or GeneXpert positive with Rifampicin sensitive at entry)

The following lists the inclusion criteria for the patients in the study:

1. New sputum-smear-positive drug-sensitive pulmonary TB patients who have not received or have received less than 10 days of anti-TB treatment (OR) sputum smear positive drug resistant pulmonary TB patients who have not received or have received less than a month of second line anti-TB treatment.
2. Age more than 18 yrs.
3. Body weight more than 30 kgs.
4. Residing within the selected TU areas.
5. Willing for study procedures, including home visits.
6. Willing to give written informed consent.

All the procedures were conducted following the guidelines of the Institutional Ethical Committee of Indian Council of Medical Research-National Institute for Research in Tuberculosis (ICMR-NIRT), Institutional Ethics Committee (IEC) No: 2016002(A). Informed and written consent for participation was obtained from all the participants involved in the study following the ethical guidelines before enrolling in the study. All the experiments conducted in the study were approved by the institutional ethical committee of ICMR-NIRT, IEC No: 2016002(A).

### Phenotypic drug susceptibility testing (DST)

Sputum samples were processed using sodium hydroxide and N-acetyl-l-cysteine (NaOH-NALC) followed by inoculating 0.5 ml of the processed specimen into a MGIT tube containing 7 ml of 7H9 broth with 0.8 ml of growth supplement (OADC-PANTA). MGIT tubes were then placed in the MGIT960 instrument until bottles flagged positive [10 υ − 10 ϖ colony forming units (CFU) per ml of medium]. Positive cultures were evaluated for contamination by inoculating a loop onto Brain Heart Infusion Agar plate and incubated at 37 °C for 48 hrs.

The immune chromatographic test (MPT64- protein Specific detection) for MTBC^65^ was used to confirm *M. tuberculosis*. Each of the positive cultures was then subjected to DST for Isoniazid (INH) and Rifampicin (RIF). To perform DST, three sets of MGIT tubes for each positive culture were used (growth control (drug free), containing Isoniazid (INH) at 0.1 µg/ml, and Rifampicin (RIF)1.0 µg/ml plus growth supplement^66^. All tubes were placed into MGIT960 instrument. Predefined algorithms of growth units between drug-free tubes (GC) and drug-containing tubes were compared by the MGIT960 system software. After 14 days, if the relative growth of the drug containing tube was less than the GC, the culture was declared as drug susceptible and if equal or exceeding GC, it was considered as drug resistant.

## DNA extraction

*M. tuberculosis* cultured on LJ medium was transferred into 1.8-ml screw-cap tubes containing ∼500 μl of TE buffer and genomic DNA extracted using the cetyltrimethyl ammonium bromide (CTAB)–NaCl extraction method, as described previously^67^. DNA purification was performed using Genomic DNA Clean and Concentrator kit (Zymo Research) according to the manufacturer’s instruction.

## Sequencing and genome analysis

DNA libraries were prepared using the NexteraXT DNA Library preparation kit (Illumina), according to the manufacturer’s instruction. Normalization of libraries was achieved by manual normalization method using analyzed library size in Bioanalyzer (Agilent), and the library quantity was measured in Qubit (Thermo Scientific). Whole genome sequencing of the 98 isolates was carried out on Illumina MiSeq instrument using the Miseq Reagent kit v3. Raw sequence reads were filtered using Trimmomatic (version 0.36) with parameters of minimum base quality and read length set to 20 and 30% of the read length^68^. Filtered reads were mapped to the reference genome H37Rv (NC_000916.3) using the Burrows-Wheeler Aligner^69^ (version 0.7.12) using the default parameters. The alignment was then corrected for InDels using picard (version 2.2.4) and GATK (version 3.5)^70^. Variants were identified using samtools (version 1.3.1) [4] and bcftools (version 1.3.1). Those variants with base quality > 50, mapping quality > 30, depth greater than 5 and at least one read in either direction were identified as high-quality variants using an in-house python script. Variants were compared against a database of mutations created by combining those reported by Bradley P *et al*.^11^, PhyResSE^15^ (as on Aug, 2016), Coll F *et al*.^12^, Farhat MR *et al*.^14^ and Desjardins CA *et al*.^13^ using an in-house python script. Lineages were identified based on a combination of lineage-defining single nucleotide polymorphisms (SNPs) reported by Coll F *et al*.^37^ and RD-analyzer^38^ that relies on the presence or absence of regions of difference specific to lineages of *M. tuberculosis*. To construct the phylogenetic tree, a pseudogenome was generated after replacing the reference base with the alternate allele identified above. Repetitive regions as reported by Holt *et al*.^71^ were masked using bedtools^72^ (v2.27.1). Pseudogenomes were used as input for SNP-Sites^73^ (Andrew J Page, *et al*., 2016, microbial genomics) to identify variable sites among the genomes. The output was then used to generate a phylogenetic tree using RAxML^74^ with GTR-GAMMA model with 1000 bootstrap replications.

### Mutation mapping, modelling and prediction of effects on *katG, inhA* and *rpoB*

Mutations identified from the sequence data were mapped on the crystal structures of *katG* (PDB ID 1SJ2), *rpoB* and *rpoC* (PDB ID 5UHC) using pyMol and the mutant models were generated using Modeller^75^. The web server of the recently updated version of SDM (Pandurangan *et al*. 2017; Worth *et al*. 2011; Topham *et al*. 1997), available at http://marid.bioc.cam.ac.uk/sdm2/ was used to predict the effects of mutations on protein structure stability. PDB files of the protein structures and text files containing the sets of mutations were provided as input to the server to estimate the change in free energy (ΔΔG) between the wild and mutant forms of the proteins. mCSM was used to investigate the effects of drug resistance mutations on protein-structure stability (http://biosig.unimelb.edu.au/mcsm/stability), protein-protein interface stability (mCSM-PPI, available at http://biosig.unimelb.edu.au/mcsm/protein_protein), and drug affinity binding using mCSM-lig (available at http://biosig.unimelb.edu.au/mcsm_lig/). PDB files of protein structures and text files containing sets of mutations were provided as inputs to the server. The PyMOL plugin Intermezzo (Ochoa *et al*., unpublished) (http://mordred.bioc.cam.ac.uk/intermezzo/) was used to calculate and visualize interatomic interactions.

## Supplementary information

Merged supplementary figures

Supplementary Dataset 1

## Data Availability

All sequences from this study have been submitted to the NCBI, Bioproject (Bioproject; https://www.ncbi.nlm.nih.gov/bioproject) under the accession PRJNA512266 and individual accession numbers for the Sequence Read Archive are given in Supplementary Table S1.
