# Supplementary material for: Identification and Characterization of Genetic Determinants of Isoniazid and Rifampicin Resistance in Mycobacterium tuberculosis in Southern India
Source: Sci Rep. 2019 Jul 16;9:10283. doi: 10.1038/s41598-019-46756-x (PMC6635374; doi:10.1038/s41598-019-46756-x)
Supplement: Supplementary file 1 — Merged supplementary figures [file 41598_2019_46756_MOESM1_ESM.pdf]

Identification and Characterization of Genetic Determinants of Isoniazid and Rifampicin Resistance in *Mycobacterium tuberculosis* in Southern India

Asma Munir[1^], Narender Kumar[2^], Suresh Babu[3^], Sembulingam Tamilzhalagan[3^], Siva Kumar S[3], Alangudi Natarajan Palaniappan[3], Dina Nair[3], Padma Priyadarshini[3], Mohan Natarajan[3], Srikanth Tripathy[3], Uma Devi Ranganathan[3], Sharon J. Peacock[2,4], Julian Parkhill[5], Tom L. Blundell[1*] and Sony Malhotra[1#*]

^Equal contribution

[1] Department of Biochemistry, University of Cambridge, Tennis Court. Rd., Cambridge, CB2 1GA, UK.

[2] Department of Medicine, University of Cambridge, Hills Rd., Cambridge, CB2 0QQ, UK.

[3]ICMR-National Institute for Research in Tuberculosis, Chennai -600031, India.

[4] London School of Hygiene & Tropical Medicine, Keppel Street, London, WC1E 7HT, UK.

[5] Wellcome Trust Sanger Institute, Hinxton, Cambridgeshire, CB10 1SA. UK

#Present address: Birkbeck College, University of London, Malet Street, WC1E7HX, UK.

*Authors for Correspondence:

Tom L. Blundell, Department of Biochemistry, University of Cambridge, Tennis Court. Rd., Cambridge, CB2 1GA, UK. Email: tom@cryst.bioc.cam.ac.uk

Sony Malhotra, Birkbeck College, University of London, Malet Street, WC1E7HX, UK. Email: s.malhotra@mail.cryst.bbk.ac.uk, sm2185@cam.ac.uk

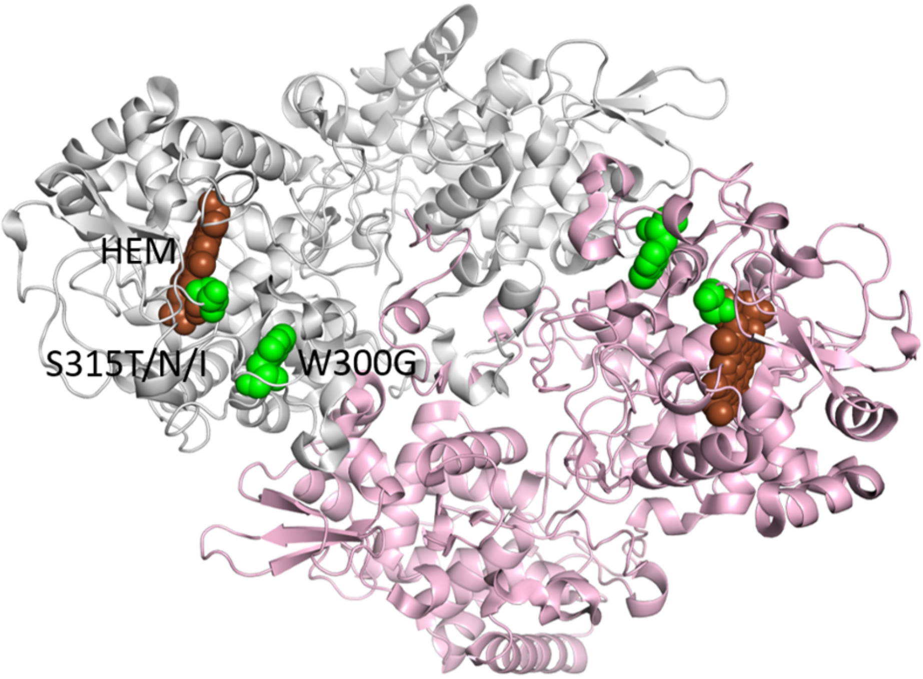

Supplementary Figure S1. Mutations (green spheres) mapped on the crystal structure of *katG*. *KatG* is a homodimer and the two protomers are shown in grey and pink. Heme is shown in brown spheres. (PDB ID: 1SJ2)

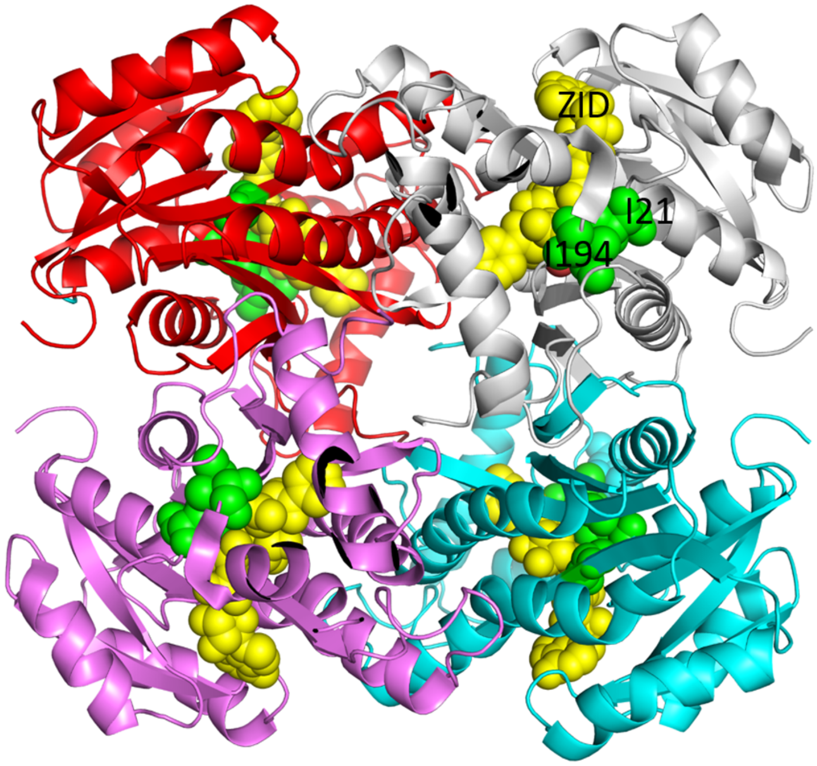

Supplementary Figure S2. Mutations mapped on the crystal structure of *inhA* (PDB ID 1ZID). The four subunits of the inhA homotetramer are coloured differently. Residues having mutations are shown in green spheres and the INH-NAD adduct (ZID, isonicotinic-acetyl-nicotinamide-adenine dinucleotide) is shown in yellow spheres.

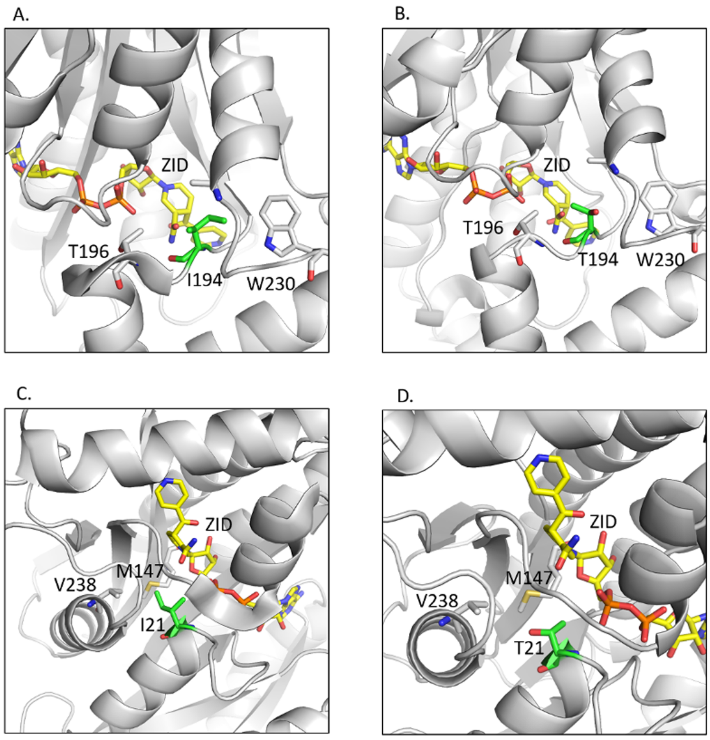

Supplementary Figure S3. A) The *inhA* wild-type residue I194 (green) is located near the INH-NAD adduct (yellow) binding site and is involved in making hydrophobic contacts with T196 and W230. B) The mutant residue T21 with a polar and shorter side-chain causes the loss of hydrophobic interactions with W230. C) The wild-type residue I21 (green) is located near the INH-NAD adduct (yellow) binding site and is involved in making hydrophobic contacts with V238 and M147. D) The mutant residue T21 with a polar side-chain causes the loss of hydrophobic interactions with V238 and M147. (.tiff)

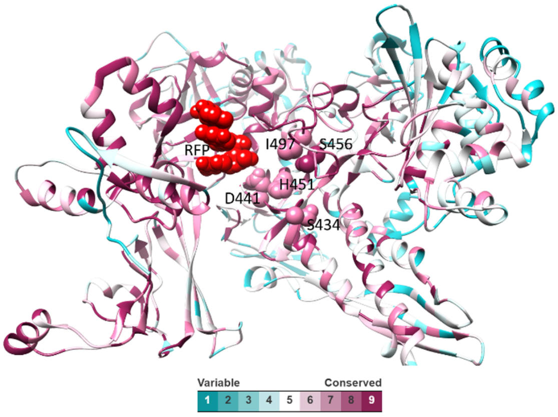

Supplementary Figure S4. Residues in *rpoB* (chain C in PDB ID 5uhc) coloured according to conservation scores calculated by ConSurf. Most rifampicin-resistant mutations occur in the highly conserved residues of the rifampicin binding pocket. (.tiff)

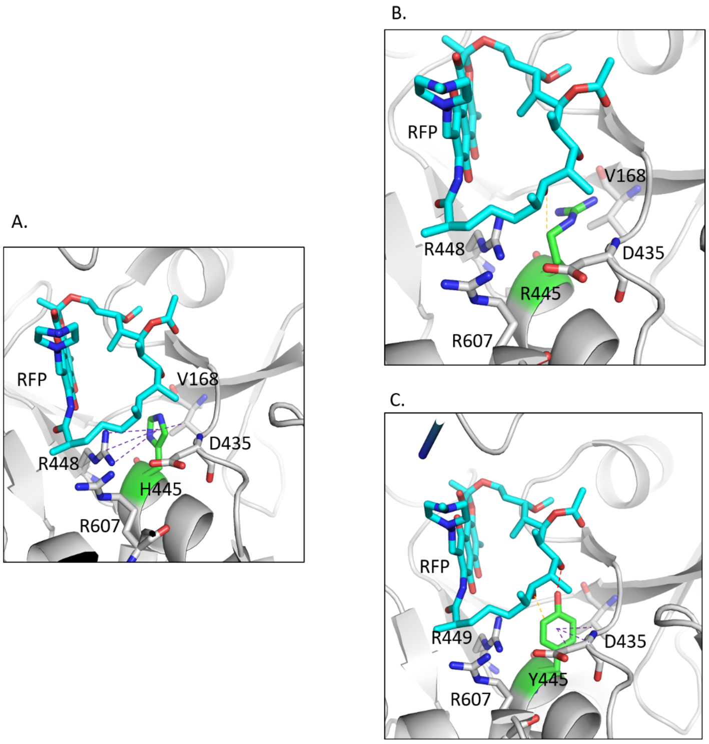

Supplementary Figure S5. A) The wildtype residue H445 in *rpoB* is involved in making carbon-pi and donor-pi interactions with R448 and V168 and hydrophobic interactions with D435. B) The mutant residue R445 loses the carbon-pi and donor-pi interactions, gains a weak hydrogen bond with RFP and causes a steric clash with D435. C) The mutant residue Y445 gains a hydrogen bond and weak hydrogen bond with RFP and carbon-pi and donor-pi interactions with D435. (.tiff)

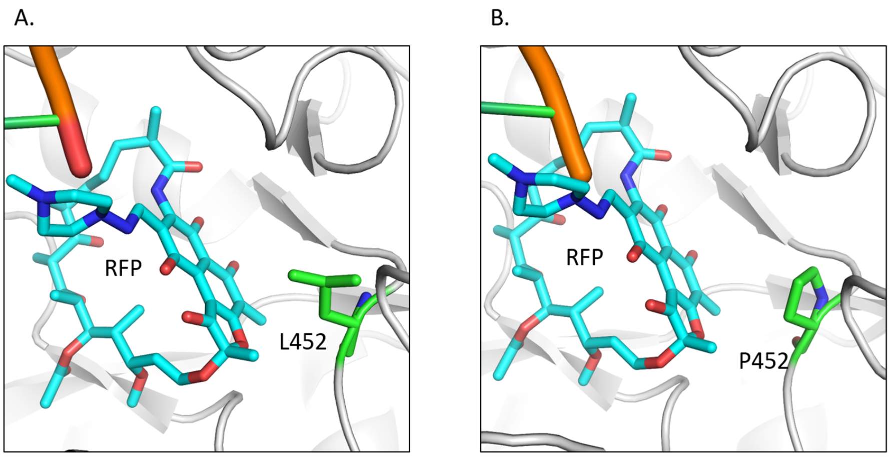

Supplementary Figure S6. A) The wild-type residue L452 in *rpoB* makes hydrophobic interactions with rifampicin. B) The mutant residue proline loses the hydrophobic interactions with the drug. (.tiff)

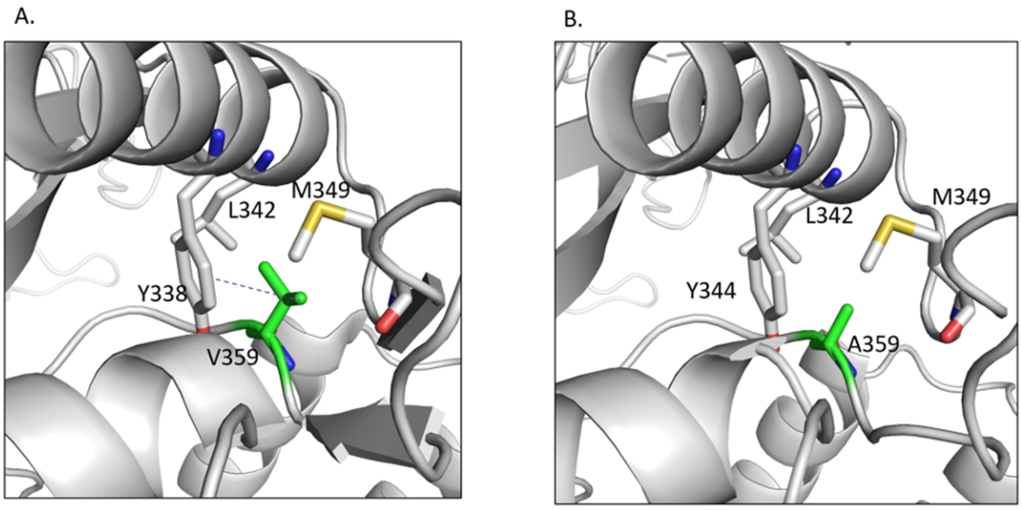

Supplementary Figure S7. The *rpoB* wildtype V359 makes carbon-pi interactions with Y338 and hydrophobic interactions with Y338, L342 and M349. B) The shorter side-chain of the mutant A359 loses carbon-pi contact with Y338 and hydrophobic interactions with L342 and M349. (.tiff)

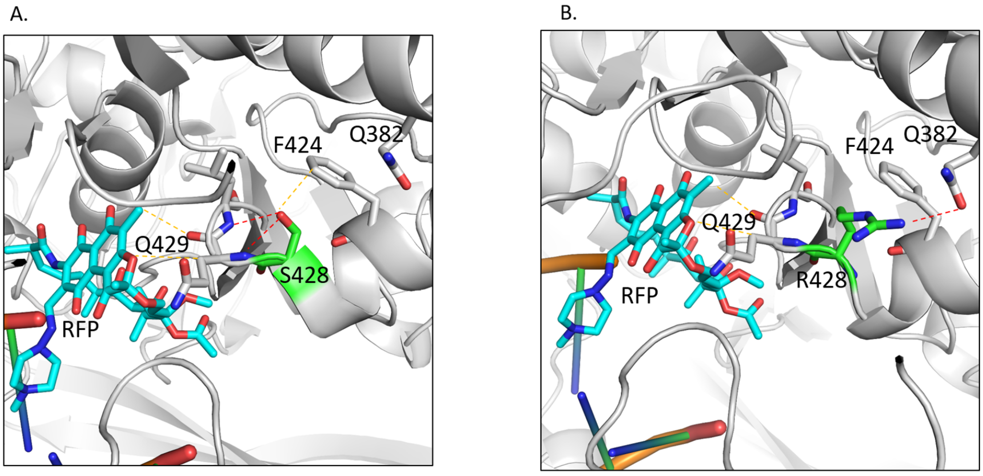

Supplementary Figure S8. A) The wildtype residue S428 in *rpoB* forms hydrogen bond with Q429 which is in turn making weak hydrogen bonds with the drug. It also forms a weak hydrogen bond with F424. B) The mutant residue R428 loses its hydrogen bonds with Q429 and and F424 and gains a hydrogen bond with Q382. (.tiff)

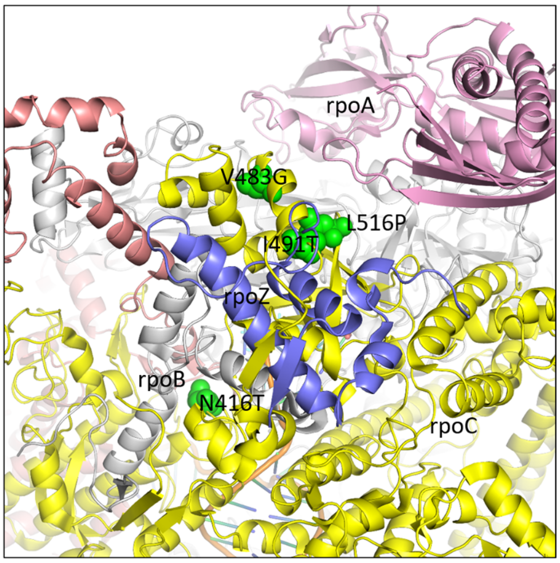

Supplementary Figure S9. Residues with mutations (green spheres) in *rpoC* are mapped on the RNA Polymerase assembly (PDB ID 5UHC). The mutations V483G, L516P, and I491T are located near the interface with *rpoA* and *rpoZ* while the mutation N416T in *rpoC* is located at the interface with *rpoB*. (.tiff)

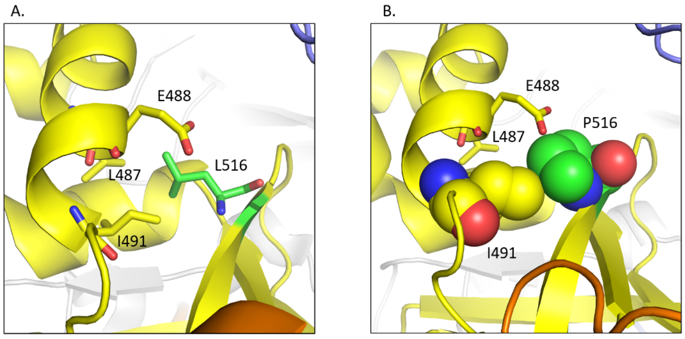

Supplementary Figure S10. A) The wild-type residue L516 in *rpoC* is involved in making hydrophobic contacts with I491, L487, E488 while B) the pyrrolidine ring of the mutated residue P516 causes a steric clash with I491. (.tiff)

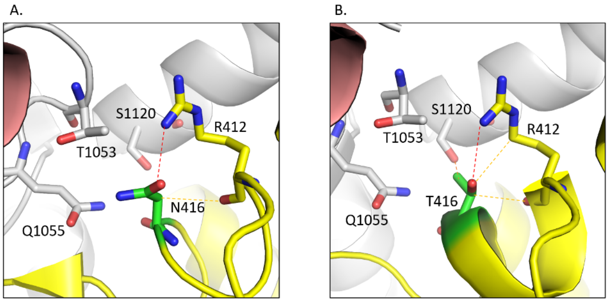

Supplementary Figure S11. A) The wild-type residue N416 in *rpoC* (yellow ribbon) forms a hydrogen bond with the side chain guanidinium NH1 of R412 and a weak hydrogen bond with the backbone of R412. B) The mutated residue T416 gains an additional weak hydrogen bond with the side chain CD of R412. It also gains a weak hydrogen bond with S1120 and hydrophobic contacts with T1053 from the *rpoB* subunit (grey ribbon). (.tiff)

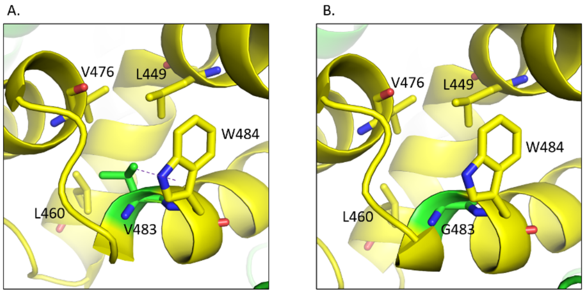

Supplementary Figure S12. A) The wild-type residue V483 in *rpoC* forms a carbon-pi contact with W484 and hydrophobic interactions with L460, V476, L449, and W484 while B) the mutated residue glycine loses all the interactions with the surrounding residues. (.tiff)

Supplementary Table S1. Sequence Read Archive accessions for the 98 samples used in this study. (.xls)
